# Supplementary material for: Multi-omic integration of microbiome data for identifying disease-associated modules
Source: Nat Commun. 2024 Mar 23;15:2621. doi: 10.1038/s41467-024-46888-3 (PMC10960825; doi:10.1038/s41467-024-46888-3)
Supplement: Supplementary file 1 — Supplementary Information [file 41467_2024_46888_MOESM1_ESM.pdf]

Supplementary notes

Supplementary note 1: Conceptual and quantitative comparison between MintTea and other related methods

As described in the main text, MintTea's objective is to identify cohesive disease-associated multi-omic microbiome modules, and as such aims to simultaneously capture both associations between omics and associations with the host disease state. Though many methods that focus on either of these two aims exist, only a few methods tackle both objectives simultaneously, each taking a unique approach to achieve these two tasks, with various, and sometime subtle, differences. In the table below, we outlined the capabilities and key objectives of several different approaches for identifying associations between the microbiome and a given disease and for pinpointing disease-associated signatures in the microbiome using multi-omic data. Importantly, in this comparison, we focused on the *conceptual* differences between these approaches, rather than on their specific performances as captured by some evaluation metric. We specifically indicated for each method whether it is explicitly designed for multi-omic data, whether it directly attempts to optimize classification ability, and whether it is easily interpretable in terms of pinpointing the most important features. We further noted whether each method also directly aims to optimize associations between the different omics (or between features from different omics). Assuming that informative features are identified by the method, we further indicated whether these features are partitioned or clustered into modules to generate distinct disease signatures, and also whether these modules are forced to include all omics (even at the cost of reduced association) or not. Lastly, given that a major limitation of many statistical and machine learning (ML) methods is their sensitivity to noisy and high-dimensional data (as is the case for microbiome data), which in turn impacts reproducibility of the obtained results, we indicated whether each method inherently incorporates mechanisms to reduce likely spurious results.

To span the space of potential approaches, we included in the comparison both ML approaches for multi-omic data, CCA extensions discussed herein (see Supplementary note 2), as well as a reasonable heuristic approach that can be used to achieve a similar goal, based on first filtering out non-informative features and then clustering the remaining features based on pairwise associations (using WGCNA for example, a highly popular method for clustering highly correlated features).

Method	Description	Specific for multi-omic data (≥ 2 omics)	Optimizes classification accuracy	Pinpoints informative features (interpretable)	Optimizes inter-omic associations	Partitions informative features into modules	Supports modules with a subset of omics[1]	Inherently controls for high-dimensional and noisy data
ML: no integration	A ML model is trained for each omic independently	✗	✓✗[2]	✓✗[3]	✗	✗	NA	✓
ML: early integration	A ML model is trained on a concatenation of all omics	✓	✓	✓✗[3]	✗	✗	✓	✓
ML: late integration	A ML model is trained for each omic independently, with a final ensemble model for final classification	✓	✓	✓✗[3]	✗	✗	✓	✓[4]
Feature selection + WGCNA[118]	Features are filtered based on disease-association, and WGCNA is applied to find correlated modules	✗	✓✗[5]	✓	✓✗	✓	✓	✓✗
sCCA / sGCCA / sMCCA[36,37]	A sparse version of classic CCA, and extensions of sCCA for ≥ 2 omics	✓	✗	✓	✓	✓	✗	✓✗[6]
DIABLO[44]	An extension of sGCCA to a supervised framework. See Ref[44]	✓	✓	✓	✓	✓	✗	✓✗[6]
MintTea	Our framework	✓	✓	✓	✓	✓	✓	✓

ML: Machine learning; CCA: Canonical correlation analysis; sCCA: Sparse CCA; sGCCA/sMCCA: sparse generalized/multiple CCA; WGCNA: Weighted correlation network analysis.
[1] As opposed to requiring each module to contain features from all omics.
[2] Does not leverage the availability of multiple omics, hence optimization is only within each omic.
[3] Depends on the selected ML algorithm and the selected explainability methods.
[4] Depends on the selected ML algorithm (e.g., whether bootstrapping/bagging mechanisms are involved).
[5] Features are filtered by their univariate association with disease. As a consequence, some of the discarded features may have actually contributed to classification accuracy when combined with others.
[6] The user must design/perform post-hoc analysis to determine the stability of components.

As evident from this comparison, only a few of these approaches directly target, as does MintTea, both aims described above. It is also important to note that while it may be possible, in principle, to first apply some ML method that optimizes predictability and then, as a second step, try to cluster informative features into modules (as in the "Feature selection + WGCNA" entry in the table above), such a two-step approach may yield different or partial modules, failing, for example, to identify features that are not sufficiently informative by themselves but that can be incorporated into informative modules to enhance predictability (as discussed in the main text).

A few other properties further differentiate MintTea from other approaches, including specifically its ability to generate modules that do not incorporate all omics (which may be crucial in future studies when the number of omics further increases), and its inherent subsampling and consensus detection procedure, which allows MintTea to better handle the high-dimensionality and noise associated with microbiome data and ultimately to provide more

conservative results. To quantify the impact of this subsampling procedure on the obtained modules' quality, robustness, and false discovery rates, we next compared MintTea's modules to those produced by the two CCA-based method listed in the above table: sMCCA (as implemented by the 'MultiCCA' function in the 'PMA' R package[36], version 1.2.1) and DIABLO[44] (Methods). For the comparisons, we used both the liver cirrhosis dataset, as it was one of the largest datasets at hand (237 samples), and the CD (Franzosa, 2019) dataset. To allow reasonable comparisons, we also tuned the penalty parameters of each method so that the average module size (or number of non-zero loadings in MultiCCA/DIABLO) is approximately 20 features (from all omics combined), with DIABLO and MultiCCA further set to output 3 modules only.

We first compared the modules in terms of their ability to classify disease and the strength of between-omic associations in unseen data. To this end, we ran each model on 70% of the samples, serving as a training set, and then computed the resulting modules on the remaining left-out 30% test set. For DIABLO and MultiCCA, the test set modules were computed using the trained loadings (feature weights), and for MintTea we took the 1st PC of all features from all omics (as done in the main text). Notably, DIABLO and MultiCCA result in one predicted variable *per omic* (3 omics in this case) that are supposedly highly correlated, and so evaluation is carried out on each such vector separately and then averaged. The entire procedure was repeated 100 times to ensure the results consistency and significance. We found that modules from all 3 methods achieved similar disease classification performance (with a notable, yet not statistically significant advantage for MintTea), with an AUC of 0.7 (standard deviation $\pm$0.14), 0.71 ($\pm$0.13) and 0.74 ($\pm$0.11) for DIABLO, MultiCCA and MintTea, respectively, in the cirrhosis datasets (Supplementary Figure S6A). In the CD datasets, AUC's were 0.71 ($\pm$0.14), 0.71 ($\pm$0.11), and 0.73 ($\pm$0.16) (Supplementary Figure S6F). Similarly, modules were comparable in their average correlation between features from different omics (Supplementary Figure S6B, S6G).

Second, we compared each method's stability when trained on different data subsets. To this end, we ran each method on two randomly selected data subsets of the same size and compared the resulting modules, assuming that more similar modules imply higher robustness of the method. The two sets of modules were compared both in terms of individual features included in the modules (Supplementary Figure S6C, S6H) and in terms of pairs of features that co-occurred in the same module in both sets (Supplementary Figure S6D, S6I). In the latter comparison, we used a z-score to quantify how likely were *pairs* of features co-occurring in

the same module in one output to also co-occur in the same module in the other output at random. The data subsets used ranged from 50% of the samples to 100%, with stability estimations increasing as subset size increased, as expected. Overall, this analysis suggests that MintTea is slightly more stable than the other methods in terms of stability of individual features selected, while in terms of stability of feature pairs, MintTea is significantly superior in one dataset, while DIABLO is superior in the other dataset, suggesting that different methods may be favored for different datasets.

Importantly, one main caveat of CCA-based methods is that, by design, they always output a set of transformations, even when no correlation structure in the data is expected to exist. When using these methods, users are thus required to carefully evaluate the obtained modules themselves, and be aware of potentially high false-positive rates especially when the number of features greatly exceeds the number of samples (again, an unfortunately common setting in microbiome studies). To demonstrate this point, we assessed the false discovery rates of all methods by applying them to a shuffled version of the data where each omic was shuffled independently (row-wise) so that any existing correlations between omics (and between omics and disease) were removed. We kept only DIABLO and MultiCCA transformations that resulted in significant between-omic correlations (FDR-corrected p-value < 0.01), and only the features in each module that had a non-redundant loading (> 0.001). Supplementary Figure S6E and S6I illustrate the results obtained over 100 random samples of 70% of the data. Evidently, even when strictly filtering the output of DIABLO/MultiCCA, their false discovery rates were still significantly higher than that of MintTea, demonstrating that MintTea is markedly more conservative and reliable.

In summary, we argue that MintTea’s dual objective, both identifying associations with disease and uncovering relationships between different omics, distinguishes it from several other supervised and unsupervised methods, and that even when compared to closely related CCA-based methods, MintTea represents a successful balance between multiple properties and metrics while maintaining substantially lower false discovery rates. Nonetheless, given the substantial variability between datasets’ characteristics, and the particular complexity of multi-omic data, it is important to acknowledge that no single method can be universally optimal, and it is strongly advisable to explore and apply a range of different methods for comprehensive analysis.

Supplementary note 2: Explanation and formulation of sCCA and its expansions

The intermediate integration approach implemented in this study is based on sparse canonical correlation analysis (sCCA) and specifically inspired by a recently introduced framework named DIABLO[44]. The modifications of sCCA which we implemented in the MintTea framework are described in the Methods section. Further explanations and formulations of sCCA and its relevant expansions are provided below.

Sparse canonical correlation analysis (sCCA)

Briefly, sCCA is a method used to identify sparse linear combinations of two sets of features describing the same set of samples, so that the resulting latent variables are maximally correlated with each other. Formally, we denote 2 standardized feature tables ('views') as $X^{(1)} \in \mathbb{R}^{n \times P_1}$ and $X^{(2)} \in \mathbb{R}^{n \times P_2}$, P_1 and P_2 being the number of features in each view respectively, and n denoting the number of samples. Sparsity is enforced using a lasso (ℓ_1) penalty function on the loading vectors, essentially making most of the loading values equal to zero, with a hyper-parameter λ determining the degree of sparsity. It is particularly helpful in cases where the number of features substantially exceeds the number of samples. sCCA then solves the following optimization problem iteratively per component (note, that the term component here refers to one set of omic-transformations) $h \in 1, \dots, H$, H being the total number of requested components (a user-defined hyper-parameter):

$$\underset{a_h^{(1)}, a_h^{(2)}}{\operatorname{argmax}} \ \operatorname{cov}\left(X_h^{(1)} \cdot a_h^{(1)}, X_h^{(2)} \cdot a_h^{(2)}\right)$$
$$s.t. \left\| a_h^{(1)} \right\|_2 = 1, \left\| a_h^{(2)} \right\|_2 = 1, \left\| a_h^{(1)} \right\|_1 \leq \lambda^{(1)}, \left\| a_h^{(2)} \right\|_1 \leq \lambda^{(2)}$$

In the above formula, $a_h^{(q)}$ is the loading vector for view q and component h, and $X_h^{(q)}$ is the residual matrix of the regression between $X_{h-1}^{(q)}$ *and* $a_{h-1}^{(q)}$. The use of residual matrices guarantees orthogonality of components within each block. We note that the use of *covariance* in the objective function, instead of *correlation* as in the original CCA, is clarified in Tenenhaus et al.[37] and is closely related to the PLS method[119].

Sparse generalized canonical correlation analysis (sGCCA) and DIABLO

To expand sCCA to $Q \geq 2$ views, i.e. generalizing sCCA, the objective function shown above is set to maximize the *sum* of correlations between all pairs of latent variables. Tenenhaus and

Tenenhaus (2011)[120] further introduced the notion of a design matrix, C, to weigh each pair of views according to the expected level of association between them, based on prior knowledge. Formally, a design matrix $C = \{c_{ij}\}$ is defined by the user, where $c_{ij} \in [0,1]\ \ \forall i,j \in 1,\ldots,Q$, so that $c_{ij} = 1$ if view i and view j are expected to be closely related, and 0 if they are expected to be unrelated. Intermediate design values, between 0 and 1, are also supported. The resulting optimization problem for sGCCA therefore becomes:

$$\underset{a_h^{(1)},\ldots,a_h^{(Q)}}{\operatorname{argmax}} \sum_{\substack{i,j=1 \\ i\neq j}}^{Q} c_{ij} \cdot \left(\operatorname{cov}\left(X_h^{(i)} \cdot a_h^{(i)},\, X_h^{(j)} \cdot a_h^{(j)}\right)\right)$$
$$s.t.\ \left\|a_h^{(q)}\right\|_2 = 1\ \ and\ \ \left\|a_h^{(q)}\right\|_1 \leq \lambda^{(q)}\ \ \forall q \in 1,\ldots,Q$$

The optimization problem is solved by an efficient iterative algorithm described in detail in Tenenhaus et al.[37]. DIABLO extends sGCCA to classification problems by adding an additional “dummy” view, namely X^{Q+1}, with one or more indicator variables representing the label of each sample (here: host disease state). By doing so, the identified “components” reflect both inter-view relationships as well as associations with the disease. Furthermore, DIABLO substitutes the lasso’s λ parameter with a “keepX” parameter indicating the exact number of variables to select from each view, for ease of use[44].

Overall, given Q view tables and a phenotype of interest, DIABLO’s sGCCA variant returns one or more “components” which are sparse linear combinations of the original views, capturing a multi-omic set of features that are both closely related with one another and jointly associated with the phenotype. Importantly, there’s a tradeoff between the two (a “correlation-discrimination tradeoff”), meaning that, at the extreme, some components may be strongly associated with the disease but have low inter-view correlations, and vice versa. Though DIABLO offers additional functionalities, mainly label prediction based on distances in the latent space, we utilized only its core sGCCA computation, as described above.

Supplementary figures

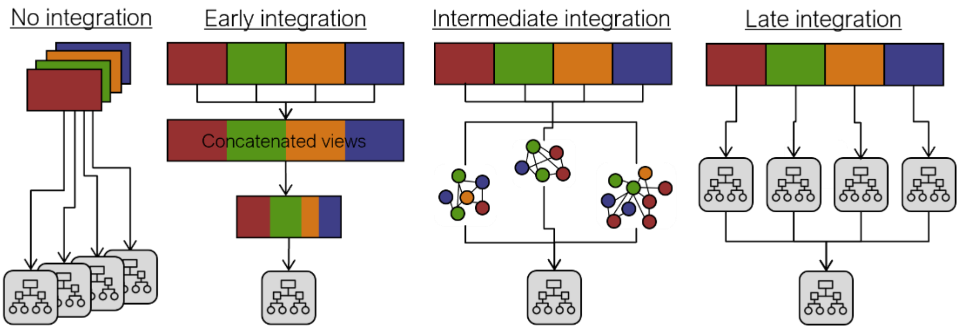

Figure S1: An illustration of different multi-view integration approaches. Different colors represent different omics.

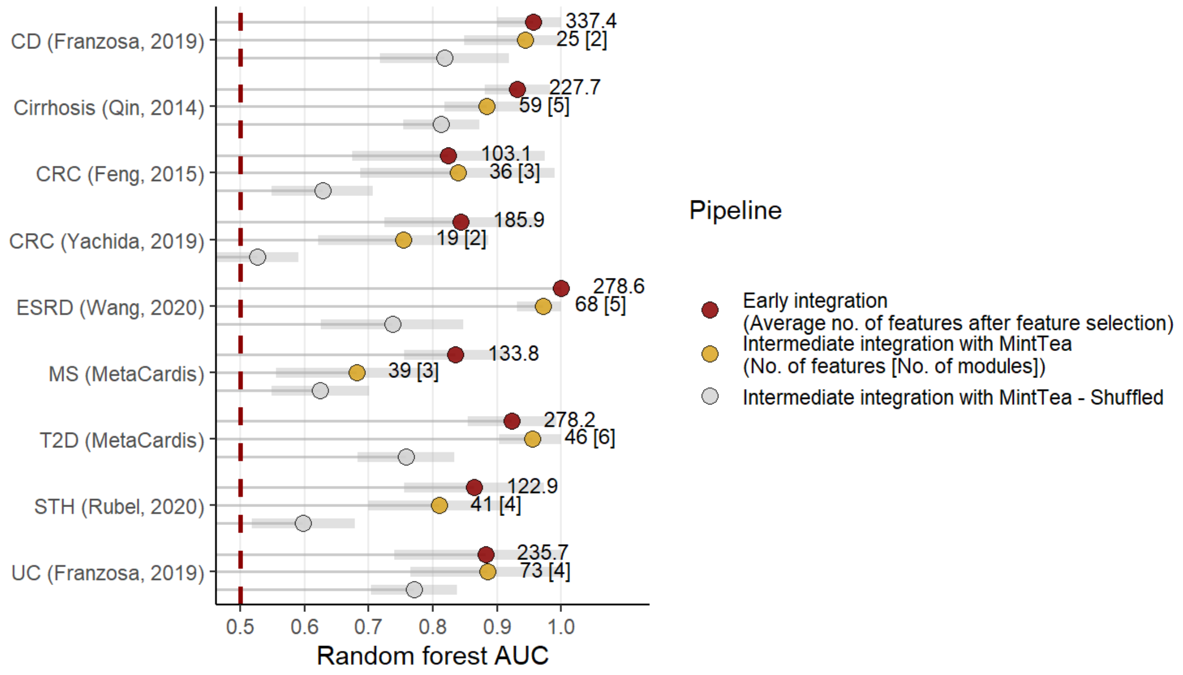

Figure S2: **Overall predictive power of multi-omic modules**. For each of the nine datasets, a random forest was trained on the first principal component of each consensus module in that dataset. Cross-validated AUC's of these models are shown in yellow, and compared to standard random forest models on raw features (in red, "early integration" approach) and models trained on random modules (grey). Numbers next to red dots represent the average number of features used for the model (averaged over cross-validation iterations). Numbers next to yellow dots represent the total number of raw features included in modules, and in square brackets the respective number of modules in that dataset.

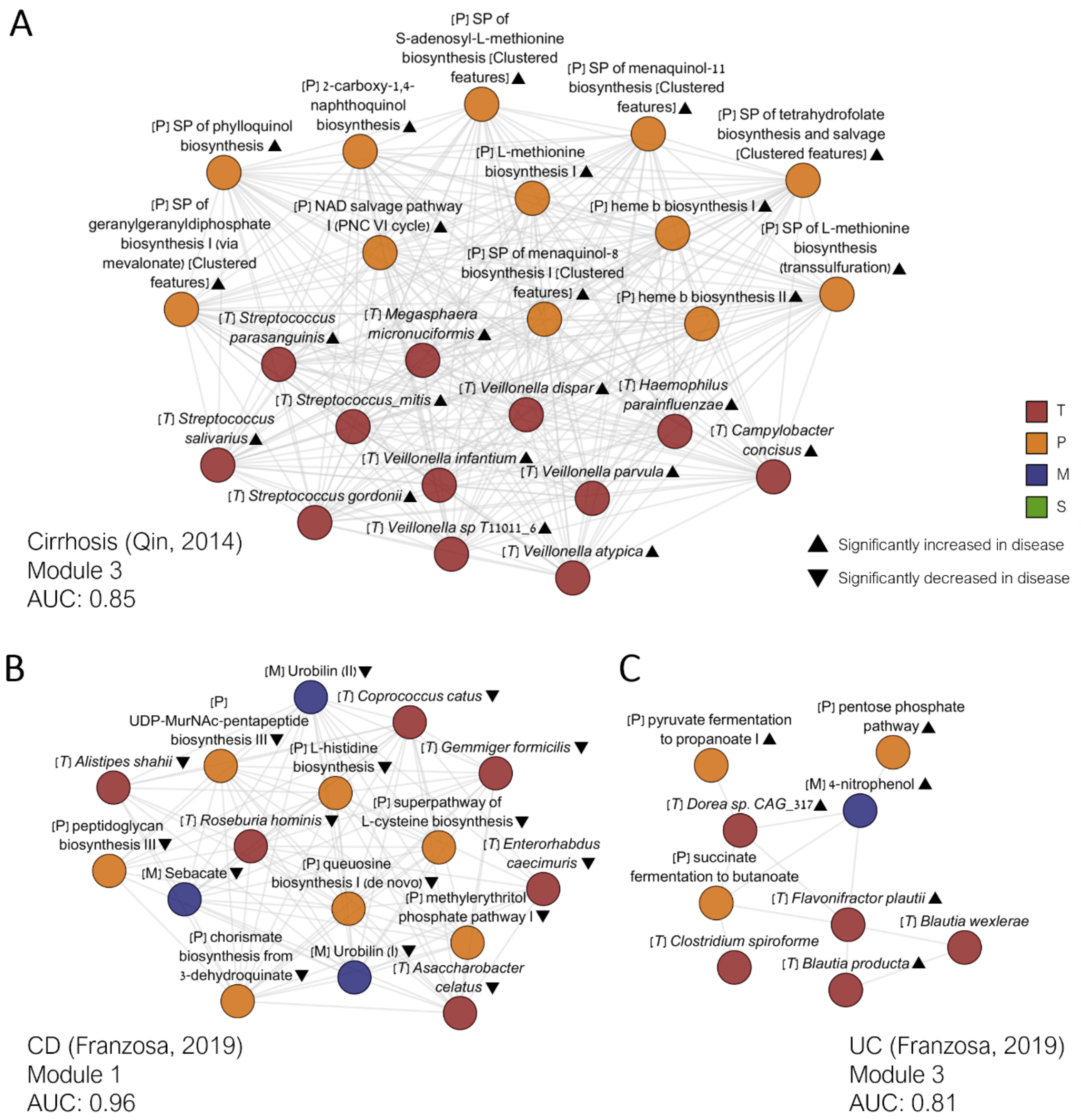

Figure S3: Additional disease-associated multi-omic modules. Colors and format are as in Figure 2D-E.

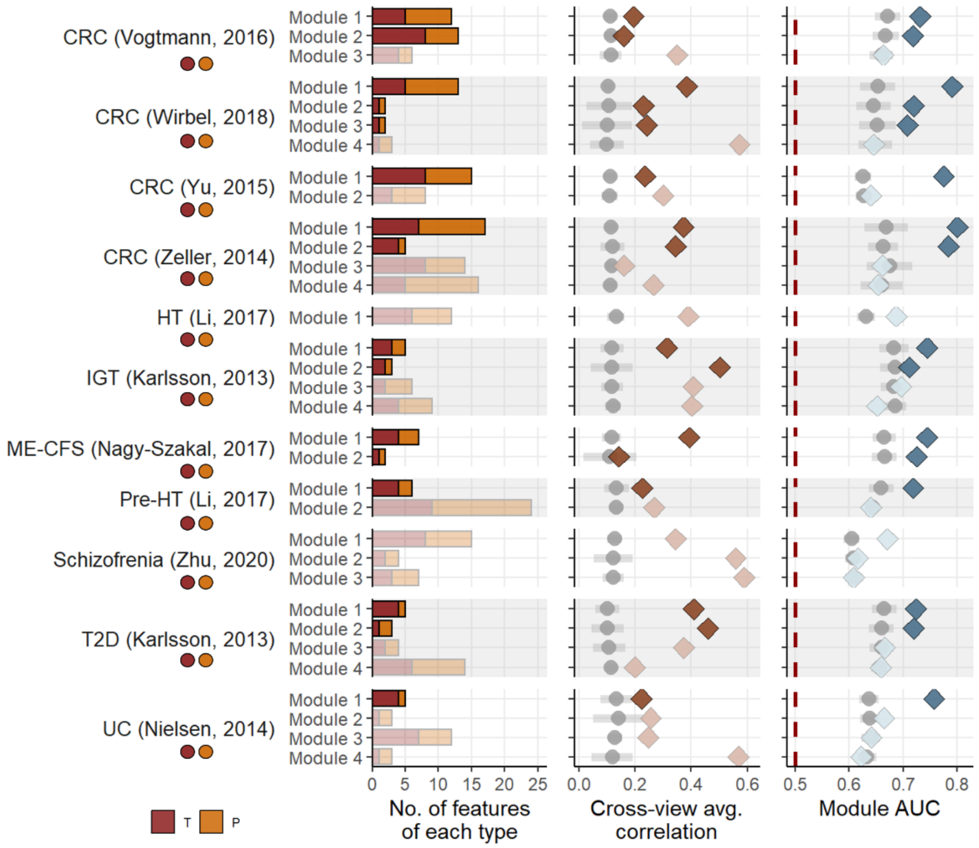

Figure S4: **Overview of MintTea modules identified in 11 additional shotgun datasets from the curatedMetagneomicData resource.** See legend of Figure 2A-C.

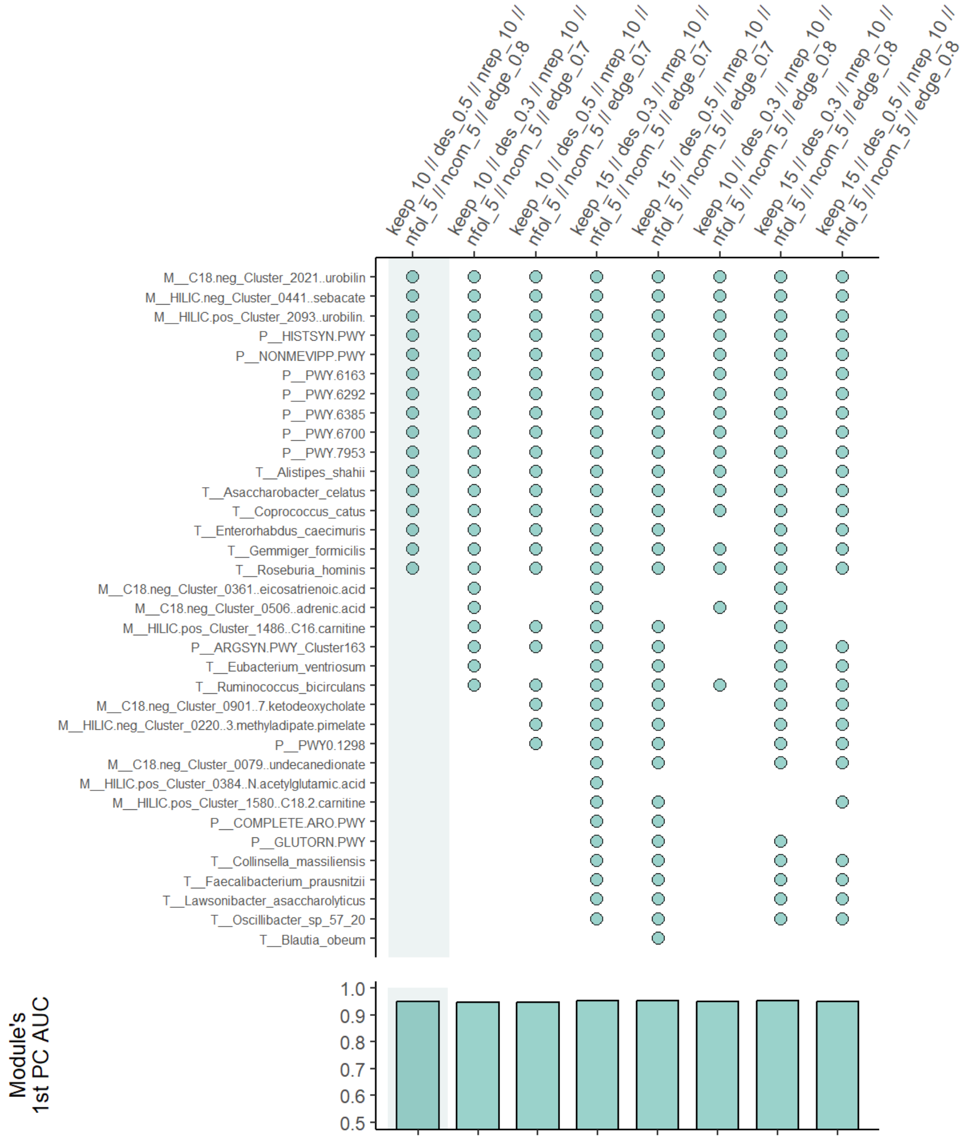

Figure S5: Example of sensitivity analysis for MintTea's pipeline parameters. The x axis lists various combinations of parameters required by our intermediate integration pipeline. A description of parameters is provided in Methods. The leftmost pipeline setting is the one used in the main results section of this study. Upper panel: Variations of module 1 from the CD (Franzosa, 2019) dataset (see also Supplementary Figure 3B), as outputted by each pipeline setting. The Y axis lists features from all omics, and points indicate that a feature was included in a multi-omic module that resulted from using that specific pipeline setting (on the x axis). Only modules overlapping with the original module (highlighted) are plotted, to illustrate how a module is generally robust to different pipeline settings. Bottom panel: Single module AUC's for each module appearing in the upper panel.

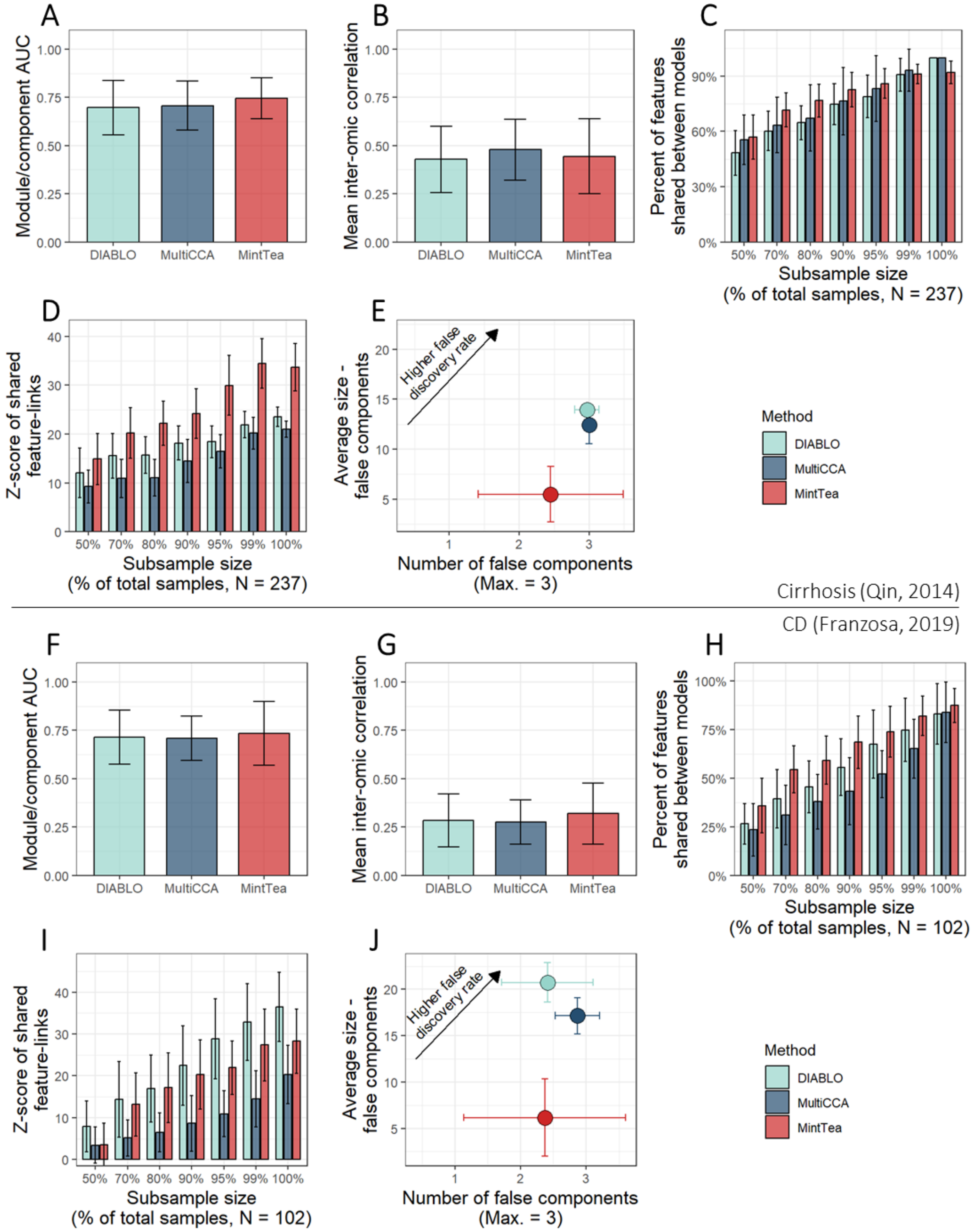

Figure S6: **MintTea comparison to DIABLO and MultiCCA**. Each panel represents a different comparison of MintTea to two other CCA-based methods: DIABLO[44] (‘mixOmics’ R package[106], version 6.18.1) and sparse multiple CCA (implemented by the MultiCCA function from the ‘PMA’ R package[36], version 1.2.1). The comparisons in panels A-E, and F-J, were made using the cirrhosis (Qin, 2014), and CD (Franzosa, 2019) datasets, respectively. (A,F) AUC of individual modules (or latent variables, in the case of DIABLO/MultiCCA)

outputted by each method. (B,G) Correlations between features from different omics, within each module (or within each set of features assigned non-zero coefficients in DIABLO/MultiCCA). (C,D,H,I) Method stability assessment. For two random data subsets (ranging from 50% of the samples to 100%, noted on the x axis), modules (or sGCCA components) were identified and compared. The more the two sets of components were similar, both in terms of individual features included in the components (C,H) and in terms of pairs of features that co-occurred in a component in both sets (D,I), we conclude that the method is more stable. See Supplementary Note 1 for details on how we computed these estimates. (E,J) False discovery rate analysis. Here, all 3 methods were applied to data that was shuffled across samples within each omic. Higher number of identified modules as well as higher numbers of features within modules indicate higher false discovery rates.

All results are averaged over 100 runs with random data subsets and error bars represent standard deviations. Penalty parameters were adjusted in each method so that the average module size (non-zero loadings in MultiCCA/DIABLO) is approximately 20 features (from all omics combined).
