# Supplementary material for: Multi-omic integration of microbiome data for identifying disease-associated modules
Source: Nat Commun. 2024 Mar 23;15:2621. doi: 10.1038/s41467-024-46888-3 (PMC10960825; doi:10.1038/s41467-024-46888-3)
Supplement: Supplementary file 3 — Description of Additional Supplementary Files [file 41467_2024_46888_MOESM3_ESM.pdf]

Description of Additional Supplementary Files

File Name: Supplementary Data S1
Description: Datasets included in the analysis

File Name: Supplementary Data S2
Description: Multi-view modules - Overview

File Name: Supplementary Data S3
Description: Multi-view modules - Detailed features

File Name: Supplementary Data S4
Description: Correlations between features within each module

File Name: Supplementary Data S5
Description: Random forest model performance results using early integration

File Name: Supplementary Data S6
Description: Random forest top feature contributors, early-integration models

File Name: Supplementary Data S7
Description: Overlapping modules across datasets

File Name: Supplementary Data S8
Description: Feature clusters
