# Supplementary material for: Multi-omic integration of microbiome data for identifying disease-associated modules
Source: Nat Commun. 2024 Mar 23;15:2621. doi: 10.1038/s41467-024-46888-3 (PMC10960825; doi:10.1038/s41467-024-46888-3)
Supplement: Supplementary file 5 — Reporting Summary [file 41467_2024_46888_MOESM5_ESM.pdf]

nature portfolio

Corresponding author(s): Elhanan Boresntein

Last updated by author(s): 2/25/2024

Reporting Summary

Nature Portfolio wishes to improve the reproducibility of the work that we publish. This form provides structure for consistency and transparency in reporting. For further information on Nature Portfolio policies, see our Editorial Policies and the Editorial Policy Checklist.

Statistics

For all statistical analyses, confirm that the following items are present in the figure legend, table legend, main text, or Methods section.

n/a	Confirmed	
☐	☒	The exact sample size (*n*) for each experimental group/condition, given as a discrete number and unit of measurement
☐	☒	A statement on whether measurements were taken from distinct samples or whether the same sample was measured repeatedly
☐	☒	The statistical test(s) used AND whether they are one- or two-sided<br>*Only common tests should be described solely by name; describe more complex techniques in the Methods section.*
☐	☒	A description of all covariates tested
☐	☒	A description of any assumptions or corrections, such as tests of normality and adjustment for multiple comparisons
☐	☒	A full description of the statistical parameters including central tendency (e.g. means) or other basic estimates (e.g. regression coefficient) AND variation (e.g. standard deviation) or associated estimates of uncertainty (e.g. confidence intervals)
☐	☒	For null hypothesis testing, the test statistic (e.g. *F*, *t*, *r*) with confidence intervals, effect sizes, degrees of freedom and *P* value noted<br>*Give P values as exact values whenever suitable.*
☒	☐	For Bayesian analysis, information on the choice of priors and Markov chain Monte Carlo settings
☒	☐	For hierarchical and complex designs, identification of the appropriate level for tests and full reporting of outcomes
☐	☒	Estimates of effect sizes (e.g. Cohen's *d*, Pearson's *r*), indicating how they were calculated

Our web collection on statistics for biologists contains articles on many of the points above.

Software and code

Policy information about availability of computer code

Data collection	R package: curatedMetagenomicData 3.2.3.
Data analysis	R-4.1.1, RStudio 2023.03.0;<br>R packages: mixOmics_6.18.1, igraph 1.3.4, lme4_1.1-27.1, dplyr_1.0.10, stringr_1.4.1, readr_2.1.2, rstatix_0.7.2, cowplot_1.1.1, ggpubr_0.4.0, ggplot2_3.4.0, tidymodels_1.0.0, ranger_0.14.1, Boruta 7.0.0. rsample 1.1.0, pROC 1.18.0.<br>GitHub repo: https://github.com/borenstein-lab/multi_view_integration_analysis<br>Additional tools: HUMAnN3 (version v3.0.1) , MetaPhlAn3 (version 3.0.14)

For manuscripts utilizing custom algorithms or software that are central to the research but not yet described in published literature, software must be made available to editors and reviewers. We strongly encourage code deposition in a community repository (e.g. GitHub). See the Nature Portfolio guidelines for submitting code & software for further information.

Data

Policy information about availability of data

All manuscripts must include a data availability statement. This statement should provide the following information, where applicable:

- Accession codes, unique identifiers, or web links for publicly available datasets
- A description of any restrictions on data availability
- For clinical datasets or third party data, please ensure that the statement adheres to our policy

Data used in this study were retrieved either from the curatedMetagenomicData package (version 3.2.3., available at: https://waldronlab.io/curatedMetagenomicData/) or from supplementary tables and deposited data from 4 specific studies (exact source of each table and accession numbers are listed in Supplementary Table S1). Functional microbiome profiles were based on metabolic pathways in either the MetaCyc (https://metacyc.org/) or KEGG (https://www.genome.jp/kegg/) databases.

Research involving human participants, their data, or biological material

Policy information about studies with human participants or human data. See also policy information about sex, gender (identity/presentation), and sexual orientation and race, ethnicity and racism.

Reporting on sex and gender	N/A
Reporting on race, ethnicity, or other socially relevant groupings	N/A
Population characteristics	N/A
Recruitment	N/A
Ethics oversight	N/A

Note that full information on the approval of the study protocol must also be provided in the manuscript.

Field-specific reporting

Please select the one below that is the best fit for your research. If you are not sure, read the appropriate sections before making your selection.

☒ Life sciences ☐ Behavioural & social sciences ☐ Ecological, evolutionary & environmental sciences

For a reference copy of the document with all sections, see nature.com/documents/nr-reporting-summary-flat.pdf

Life sciences study design

All studies must disclose on these points even when the disclosure is negative.

Sample size	No sample size calculation was conducted. Sample sizes were determined by the original studies from which we obtained the omic data. Cross-validation of results was performed, when relevant, to validate the robustness of results.
Data exclusions	In the data from Yachida et al., patients with early-stage colorectal cancer were excluded as detailed in the manuscript and further described in Supplementary Table 1. In the data from the MetaCardis cohort, we excluded patients with recent antibiotics intake or multiple drugs intake, as detailed in the manuscript and in Supplementary Table 1. No patients were excluded from the other cohorts.
Replication	We ensured reproducibility through cross-validation, particularly in assessing Area Under the ROC Curve (AUC). AUC's reported in the manuscript are calculated on samples unseen during training. Replication in completely independent datasets was not feasable due to variations in exact omics availability and technology used.
Randomization	N/A. Our study leverages publicly available cohorts and data, thus the concepts of randomization and blinding are not applicable as the analysis is conducted computationally on pre-existing datasets without intervention or human subjectivity.
Blinding	N/A. Our study leverages publicly available cohorts and data, thus the concepts of randomization and blinding are not applicable as the analysis is conducted computationally on pre-existing datasets without intervention or human subjectivity.

Reporting for specific materials, systems and methods

We require information from authors about some types of materials, experimental systems and methods used in many studies. Here, indicate whether each material, system or method listed is relevant to your study. If you are not sure if a list item applies to your research, read the appropriate section before selecting a response.

Materials & experimental systems

n/a	Involved in the study
☒	☐ Antibodies
☒	☐ Eukaryotic cell lines
☒	☐ Palaeontology and archaeology
☒	☐ Animals and other organisms
☒	☐ Clinical data
☒	☐ Dual use research of concern
☒	☐ Plants

Methods

n/a	Involved in the study
☒	☐ ChIP-seq
☒	☐ Flow cytometry
☒	☐ MRI-based neuroimaging
